# Removal of Uranium by Polymer Metal Oxide Nanofiber Composites: Enhanced Performance through Integration of Phthalic Acid

**DOI:** 10.1021/acsaenm.5c00648

**Published:** 2025-10-17

**Authors:** Sewoon Kim, Sarah K. Scherrer, Nicole M. Shapiro, Chang Min Park, Tori Z. Forbes, David M. Cwiertny

**Affiliations:** † Department of Civil and Environmental Engineering, 4083 University of Iowa, Iowa City, Iowa 52242, United States; ‡ Department of Chemistry, University of Iowa, Iowa City, Iowa 52242, United States; § Department of Civil and Environmental Engineering, Kyungpook National University, 80 Daehak-ro, Buk-gu, Daegu 41566, Republic of Korea

**Keywords:** metal oxide, uranium, electrospinning, organic acid, uptake

## Abstract

We developed polyacrylonitrile (PAN) nanofibers embedded with various commercially available metal oxide particles (Fe_2_O_3_, TiO_2_, MnO_2_, Co_3_O_4_, CoFe_2_O_4_, ZnFe_2_O_4_) via electrospinning for the removal of uranium (U­(VI)) from aqueous systems. We compared the performance of composites electrospun with and without the inclusion of phthalic acid (PTA), building on our prior evidence that PTA can promote particle dispersion in precursor sol–gels. Characterization using SEM, TEM, XPS, and BET confirmed that PTA results in improved metal oxide distribution within the polymer fiber, promotes enrichment of metal oxides on the nanofiber surface, and increases composite surface area and porosity. In batch sorption experiments, PTA-containing composites consistently exhibited greater U­(VI) uptake than those without PTA, producing more than 2- and 3-fold increases for top-performing Fe_2_O_3_ and TiO_2_ composites, respectively. For Fe_2_O_3_ and TiO_2_ composites with PTA, U­(VI) uptake increased from pH 2 to 7, suggesting a contribution from retained phthalic acid (as phthalate), and isotherm studies revealed sorption capacities exceeding ∼8 mg U/g (corresponding to >90% of U­(VI) removal) at environmentally relevant concentrations (∼1 μM). These composites are offer a simple, one-pot fabrication route to high-performing U­(VI) sorbents in which PTA improves metal oxide distribution, surface area and pore volume of the polymer–metal oxide composites while also contributing to U­(VI) uptake via cooperative binding with embedded metal oxides.

## Graphical abstract

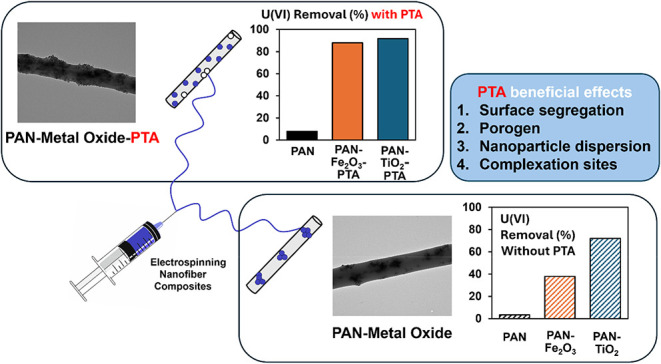

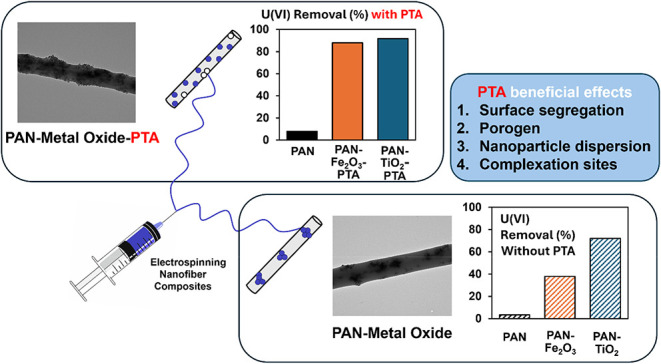

## 1 Introduction

Uranium contamination of groundwater resources remains a public health challenge in many parts of the United States, particularly in remote communities of the arid Southwest where access to alternative water sources is limited.^1,2^ Treatment for uranium removal is a viable option to improve water quality, with materials-based approaches being most common.^3^ These include the use of strong base anion exchange resins^4,5^ and reverse osmosis membranes,^6,7^ which represent two commonly used technologies for uranium removal during drinking water treatment. However, these technologies are not without their limitations; both produce a concentrated waste brine enriched with uranium that can be difficult to dispose of, and reverse osmosis is expensive and energy intensive.^8^

In search of new technologies for uranium treatment, researchers have explored the use of nanomaterial-based sorbents owing to their large specific surface area and high inherent surface activity.^9–11^ Many materials, including naturally abundant species like iron oxides, exhibit an affinity for uranium uptake from water across broad ranges of pH relevant to drinking water.^12^ Indeed, a common form of hexavalent uranium [U­(VI)] in many natural waters is the uranyl cation (UO_2_^2+^), which would be susceptible to sorption onto negatively charged surfaces that are common of metal oxides at circumneutral pH. For example, Zhao et al.^13^ reported that U­(VI) sorption using Fe_2_O_3_ (hematite) was studied across a pH range of 3 to 10. The results exhibited a maximum U­(VI) sorption capacity (*q*_max_) of 5.59 mg/g at pH 5.5. Similarly, Wang et al.^14^ investigated U­(VI) sorption using TiO_2_ over a pH range of 2 to 11. They observed a gradual increase in sorption capacity, reaching a *q*_max_ of 10 mg/g at pH values above 4.

Recently, we have demonstrated how polymer composites of nanoscale iron oxides can be synthesized via electrospinning to yield high-performing materials enriched in iron oxide sorption sites at or near the polymer surface.^15,16^ We found that addition of organic acid additives including simple organic acids to electrospinning sol–gels (i) improves the dispersion of iron oxide particles in the polymer precursor sol–gel; (ii) promotes surface segregation (i.e., preferential surface accumulation) of the iron oxides during electrospinning, and (iii) produces composites with higher porosity through the eventual release of some fraction of the organic acid (i.e., the acid acts as a porogen). Accordingly, for sorption of dissolved lead, we found that electrospun composites of nanoscale iron oxides embedded within polyacrylonitrile (PAN) nanofibers exhibited roughly 4-fold greater sorption capacity after integration of as little as 3 wt % of phthalic acid (PTA) to the electrospinning precursor solution. This simple, “one-pot” synthesis to produce surface-enriched composites greatly increases the versatility of electrospinning, which is an industrially viable and scalable fabrication technology for nanofiber-based materials.^17^

Here, we explore how the additive PTA influences the synthesis and performance of polymer composites made from various metal oxides that are known or suspected to be active toward U­(VI). Commercially available metal oxide particles ranging from nano- to micron-scale including Fe_2_O_3_, TiO_2_, MnO_2_, Co_3_O_4_, CoFe_2_O_4_, and ZnFe_2_O_4_ were incorporated into PAN nanofibers via a sol–gel precursor method, with sol–gels prepared both with and without addition of PTA. Characterization of the synthesized nanofiber composites included analysis of the metal oxide crystal phase, particle size, surface charge, morphology, and surface chemistry. Sorption experiments were conducted to evaluate the U­(VI) uptake performance of the nanofiber composites under varying initial U­(VI) concentrations and solution pH. Overall, we found that the addition of PTA increases the uptake of U­(VI) by some, but not all, metal oxide-PAN composites, suggesting that PTA-metal oxide interactions within the sol–gel are likely critical to increasing U­(VI) uptake. The insights gained from this work will help to advance the development of low-cost, efficient and sustainable strategies for remediation of U­(VI), as well as other heavy metals, from aqueous environments including drinking water sources and waste streams.

## 2 Experimental Section

### 2.1 Reagents

Details about all chemical reagents are provided in the Supporting Information (SI).

### 2.2 Metal Oxide Sorbents

We worked with several metal oxides and mixed metal oxides that were suspected to be suitable sorbents for U­(VI) based on prior reports for materials with similar composition.^18,19^ These were hematite (α-Fe_2_O_3_), titanium dioxide (TiO_2_), manganese oxide (MnO_2_), cobalt oxide (Co_3_O_4_) and mixed metal oxides of iron with cobalt (CoFe_2_O_4_) and zinc (ZnFe_2_O_4_). All oxides were purchased commercially and used in sorption studies and composites as received. Additional details can be found in Table S1.

### 2.3 Electrospun Nanofiber Fabrication

To prepare electrospinning sol–gel precursor solutions, 25 wt % of metal oxide powders and 0 or 30 wt % of PTA were first dissolved and dispersed in dimethylformamide (DMF) via sonication for 5 h. We note that all wt % values are provided relative to the mass of PAN used in composite fabrication. PAN was then added to the metal oxide or metal oxide-PTA suspension and mixed at 60 °C for 2 h to produce a homogeneous sol–gel suspension. The resulting sol–gel was then left at room temperature for at least 8 h. During this time, it was gently shaken to maintain homogeneity. Afterward, the sol–gel was loaded into a 12 mL plastic syringe and mounted onto a syringe pump for electrospinning. The syringe was connected to polyethylene tubing, the other end of which was connected to a metal nozzle adapter and a 23-gauge syringe needle. The needle tip was positioned 10 cm from a rotating metal drum collector that was covered in Al foil and grounded.

During electrospinning, the sol–gel was ejected from the syringe needle at a pumping rate of 0.5 mL/h. Other electrospinning parameters included a collector rotation speed of ∼500 rpm, 15 kV of applied voltage at the syringe needle tip, and a chamber atmosphere with 20% relative humidity and a temperature of 20 °C. After completion of the electrospinning, the deposited layer of nanofiber composite was removed from the collector surface and rinsed extensively with deionized water prior to characterization and performance testing. Details of characterization methods and composite rinsing procedures are described in the SI.

### 2.4 Batch Sorption Experiments

The U­(VI) stock was created from a 100 ppm (ppm) standard U solution (1% v/v nitric acid matrix) purchased from Inorganic Ventures (Lot: N2–U666947). For the sorption experiments, a 5 μM U­(VI) stock solution was made by diluting with a 5 mM CaCO_3_ buffer. The solution was adjusted to the target pH value, allowed to equilibrate for one month and then used in subsequent uptake experiments.

All batch experiments were conducted in triplicate at room temperature and in aqueous solutions at pH 7 buffered by 5 mM CaCO_3_. All U­(VI) sorption studies were conducted with a fixed sorbent loading of either 0.25 g of metal oxide powder or nanofiber composites per liter. All experiments were carried out using polypropylene centrifuge tubes and caps to avoid loss of U­(VI) via sorption to glass. Sorption isotherms were carried out by varying the initial concentration of U­(VI) from 0.1 to 10 μM, where 24 h of contact time was found to be sufficient to achieve sorption equilibrium. pH edge experiments were conducted to evaluate uptake as a function of solution pH, using reactors and procedures identical to those used in batch sorption isotherm experiments. The U­(VI) concentration was fixed at 1 μM, with a nanofiber composite dosage of 0.25 g/L. Solution pH was adjusted using either 1 M NaOH or HNO_3_ to the desired starting pH in the 5 mM CaCO_3_ buffer system.

### 2.5 Analytical Methods

Uranium concentrations were determined using inductively coupled plasma mass spectrometry (ICP-MS). Details associated with this analysis including sample processing and instrumentation are described in the SI.

## 3 Results and Discussion

### 3.1 Characterization of Metal Oxide Particles

The crystalline properties of commercially available Fe_2_O_3_, TiO_2_, MnO_2_, Co_3_O_4_, CoFe_2_O_4_, and ZnFe_2_O_4_ were confirmed by XRD analysis as shown in Figure S1. All selected metal oxide particles were consistent with expectations from their vendor based on reference crystal phases available in JADE Software. For example, Fe_2_O_3_ corresponded to hematite (α-Fe_2_O_3_), TiO_2_ exhibited a mix of anatase and rutile phases, MnO_2_ was consistent with pyrolusite, and ZnFe_2_O_4_ aligned with franklinite.

Figure S2 shows transmission electron microscopy (TEM) images of each commercially available metal oxide powder from which the primary particle size of each material was estimated by measuring at least *n* = 100 particles. Fe_2_O_3_ and MnO_2_ particles exhibited the smallest [5 (±2) nm] and biggest [4 (±0.2) μm] average particle size, respectively. The remaining particles were in the range of 30–55 nm. All sizes were generally consistent with expectations from vendors. Although there is a rather broad distribution in the size of MnO_2_ particles, including some fraction below 1 μm in dimension, we would anticipate the MnO_2_ particles would be more difficult to integrate into electrospun PAN fibers. Based on our prior work, PAN nanofibers are typically most easily synthesized on the order of 100 to several 100 s of nm in average diameter,^15^ which is smaller than the particle size of most MnO_2_ particles in this commercial material.

Surface charge on the particles can play an important role in sorption of U­(VI) by driving electrostatic interactions with dissolved U­(VI) species. As a measure of particle surface charge, Figure S3 shows zeta potential values as a function of pH for suspensions of each metal oxide. Zeta potential values for all metal oxide suspensions decreased with increasing solution pH, as is to be expected with deprotonation of surface hydroxyl groups.^20,21^ Notably, at pH 7, which is the pH value used in the majority of our U­(VI) uptake experiments, the zeta potential values (in mV) for each metal oxide suspension were as follows, from most negative to most positive: TiO_2_ (−22) < Fe_2_O_3_ (−11) < Co_3_O_4_ (−1) < CoFe_2_O_4_ (−0.1) < MnO_2_ (+13) < ZnFe_2_O_4_ (+13). Based on these pH-dependent measurements, we estimate the pH value at which the surface exhibits a net zero charge (i.e., pH_zpc_) is ∼4 for Fe_2_O_3_ and TiO_2_, ∼7 for Co_3_O_4_ and CoFe_2_O_4_, ∼8 for ZnFe_2_O_4_, and ∼9 for MnO_2_. These values generally align with previous reports for suspensions of these metal oxides. For example, previous studies have reported pH_zpc_ values of Fe_2_O_3_ and TiO_2_ around 4 ^22^ and 6,^23^ respectively.

### 3.2 U­(VI) Uptake in Suspensions of Metal Oxide Particles

Figure 1 compares U­(VI) sorption of suspensions of each metal oxide particle (0.25 g/L) initially exposed to 1 μM of total U­(VI) at pH 7. Data are shown for sorbed U­(VI) concentrations (*q*_e_) over time, based on the difference in the initial concentration of dissolved U­(VI) and the concentration remaining in solution after 24 and 48 h. Equilibrium U­(VI) uptake was achieved with all metal oxides by 24 h, with comparable sorbed U­(VI) concentrations after 48 h. The highest U­(VI) sorption was observed for TiO_2_, ZnFe_2_O_4_, and CO_3_O_4_, with uptake between ∼0.4 and 0.45 mg/g. Considerably lower uptake was observed for Fe_2_O_3_, closer to 0.1 mg/g, and the lowest capacity was exhibited by MnO_2_ at around 0.02 mg/g.

**Figure 1:**
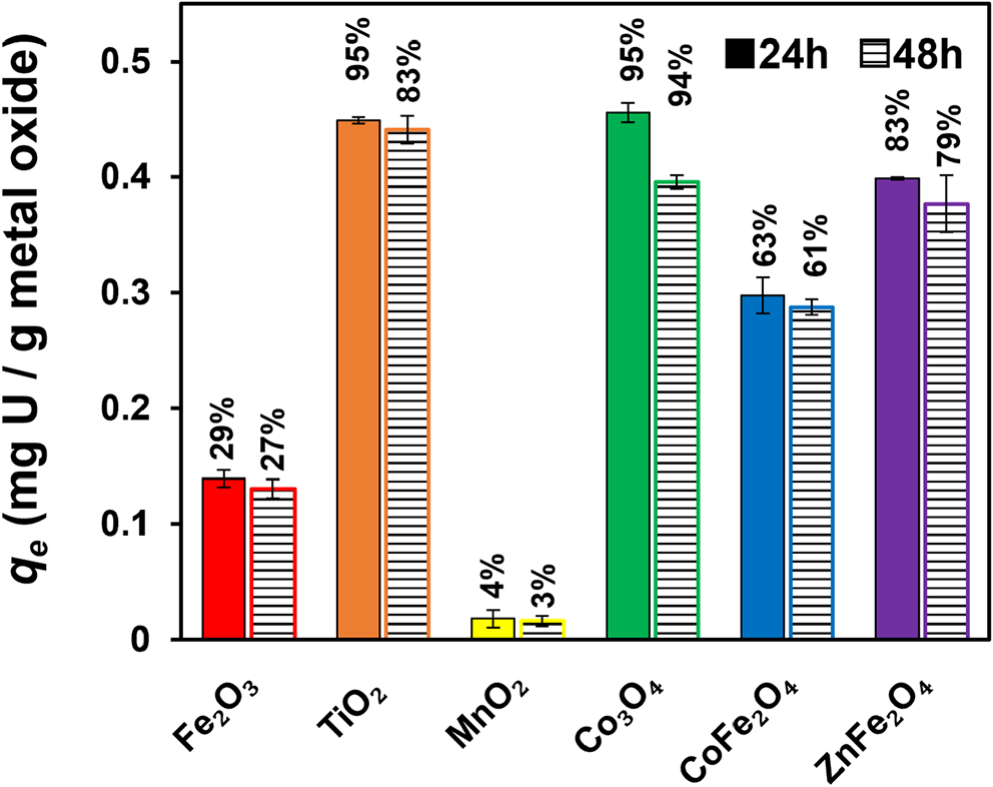
Sorbed U­(VI) mass at equilibrium (*q*_e_) in various metal oxide suspensions after 24 and 48 h. Numbers indicate the percentage of total U­(VI) removed in each suspension, with values approaching 100% indicating near complete uptake. Error bars represent one standard deviation from triplicate experiments. Experimental conditions: initial U­(VI) concentration of 1 μM, pH 7 (buffered using 5 mM CaCO_3_), and metal oxide loading of 0.25 g/L.

There are no immediately obvious trends in U­(VI) uptake based simply on our characterization of metal oxide particles and their suspensions. For example, at pH 7, the most negative zeta potential values were observed for TiO_2_ and Fe_2_O_3_, although TiO_2_ exhibited nearly 4-fold greater uptake than Fe_2_O_3_. Similarly, we measured identical zeta potential values for MnO_2_ and ZnFe_2_O_4_ suspensions at pH 7, although ZnFe_2_O_4_ resulted in considerably greater (nearly 20-fold) U­(VI) uptake than MnO_2_. Although the large particle size of MnO_2_ results in lower specific surface area that likely limits U­(VI) uptake, the largest available surface area materials, 5 nm Fe_2_O_3_ particles, resulted in the second lowest extent of U­(VI) uptake. We conclude, therefore, that specific interactions between dissolved U­(VI) species and surface binding sites are primarily responsible for the relative extent of U­(VI) uptake on the different metal oxides.

Indeed, trends in U­(VI) sorption across the different metal oxide suspensions can be better rationalized based on the speciation of U­(VI) under our experimental conditions [see the speciation diagram for the U­(VI) stock solution in Figure S4] and prior sorption studies with analogous metal oxides. At pH 7, the major aqueous U­(VI) complexes include the [Ca_2_UO_2_(CO_3_)_3_]^0^ and [UO_2_(CO_3_)_2_]^2–^ representing 31 and 52% of the total U, respectively, with both [UO_2_(CO_3_)_3_]^4–^ and [UO_2_CO_3_]^0^ as additional minor species. The formation of carbonate complexes does influence the uptake behavior of the U­(VI) as Duff and Amhrein^24^ have previously demonstrated that it contributes to lower uptake on soil and mineral surfaces. The 30% uptake observed for the Fe_2_O_3_ nanoparticles does match well with previous results from Bargar et al.,^25^ who observed 20 and 30% uptake onto hematite surfaces at pH 4 and 8.9, respectively. The authors linked this uptake back to metal-bridging ternary hematite-U­(VI)-carbonato complexes with similarities to the [UO_2_(CO_3_)_2_]^2–^ species. The low sorption observed for the MnO_2_ material was somewhat surprising given that Liu et al.^26^ previously demonstrated U­(VI) uptake onto MnO_2_ phases. However, they also noted that the pyrolusite phase (β-MnO_2_) exhibited the lowest U­(VI) uptake capacity of all the polymorphs, and Wang et al.^27^ noted that the formation of U­(VI) carbonate species significantly decreased the U uptake in batch experiments, supporting the results observed herein. High U­(VI) uptake was previously observed for TiO_2_ with only minimal effects of carbonate at pH 7.^28^ Less is known regarding uptake on Co_3_O_4_ and the ferrite phases, however U­(VI) uptake in the current study is within range of previously reported spinel and ferrite compounds.^29,30^

### 3.3 Characterization of PTA-Assisted Nanofiber Composites

Optical images of the synthesized composite fibers highlight the different colors corresponding to each metal oxide (Figure S5­(a)), consistent with metal oxides being embedded into the PAN fibers during synthesis. The colors of the composite layers largely matched expectations from the color of the metal oxide powders used to prepare the different composite sol–gels. To the naked eye, there were no observable differences in the appearance of composites fabricated with and without PTA. However, there were observable differences during synthesis of formulations with and without PTA, especially for Fe_2_O_3_ and TiO_2_. For these oxides, addition of PTA to the electrospinning sol–gel resulted in improved dispersion of the metal oxides. This was best observed by differences in the degree of particle settling that occurred over long time scales (longer than those associated with composite fabrication) in these sol–gels (see Figure S5­(b)).

We hypothesize that interactions between the PTA and select metal oxides results in improved particle dispersion in the sol–gel. The PTA appears to be acting as a stabilizing or capping ligand on the oxide surface, thereby limiting aggregation and improving the distribution of the metal oxides in the resulting composite fibers. PTA is a diprotic acid with reported p*K*_a_ values of 2.9 and 5.1 at 25 °C in water.^31^ The electrospinning precursor sol–gel solution is prepared in DMF and contains 9 wt % of PAN monomer. Accordingly, we are unable to apply aquatic p*K*a values to these nonaqueous mixtures. Nevertheless, visual evidence of more stable oxide particles, as shown in Figure S5­(b) for Fe_2_O_3_ and TiO_2_, suggests some degree of PTA interaction with select oxides in this DMF/PAN mixture

Our hypothesis of improved metal oxide distribution in PTA-containing composites is supported by microscopic characterization of the electrospun composite fibers. Figure 2 shows representative scanning electron microscopy (SEM) and TEM images of PAN-Fe_2_O_3_, PAN-TiO_2_, and PAN-MnO_2_ nanofibers fabricated with and without the addition of PTA to the electrospinning sol–gel. For PAN-Fe_2_O_3_ and PAN-TiO_2_, SEM imaging revealed relatively rough nanofiber surfaces, presumably resulting from the embedded metal oxide particles (and/or their aggregates) at or near the fiber surface. Corresponding TEM images illustrate the distribution of Fe_2_O_3_ and TiO_2_ nanoparticles within the PAN fibers, and generally we observed a more uniform distribution of nanoparticles throughout the fibers when composites were prepared with PTA. We acknowledge that because of the nature of TEM it is difficult to ascertain whether the Fe_2_O_3_ and TiO_2_ nanoparticles are located near the surface or embedded deeper within the polymer fibers.

**Figure 2:**
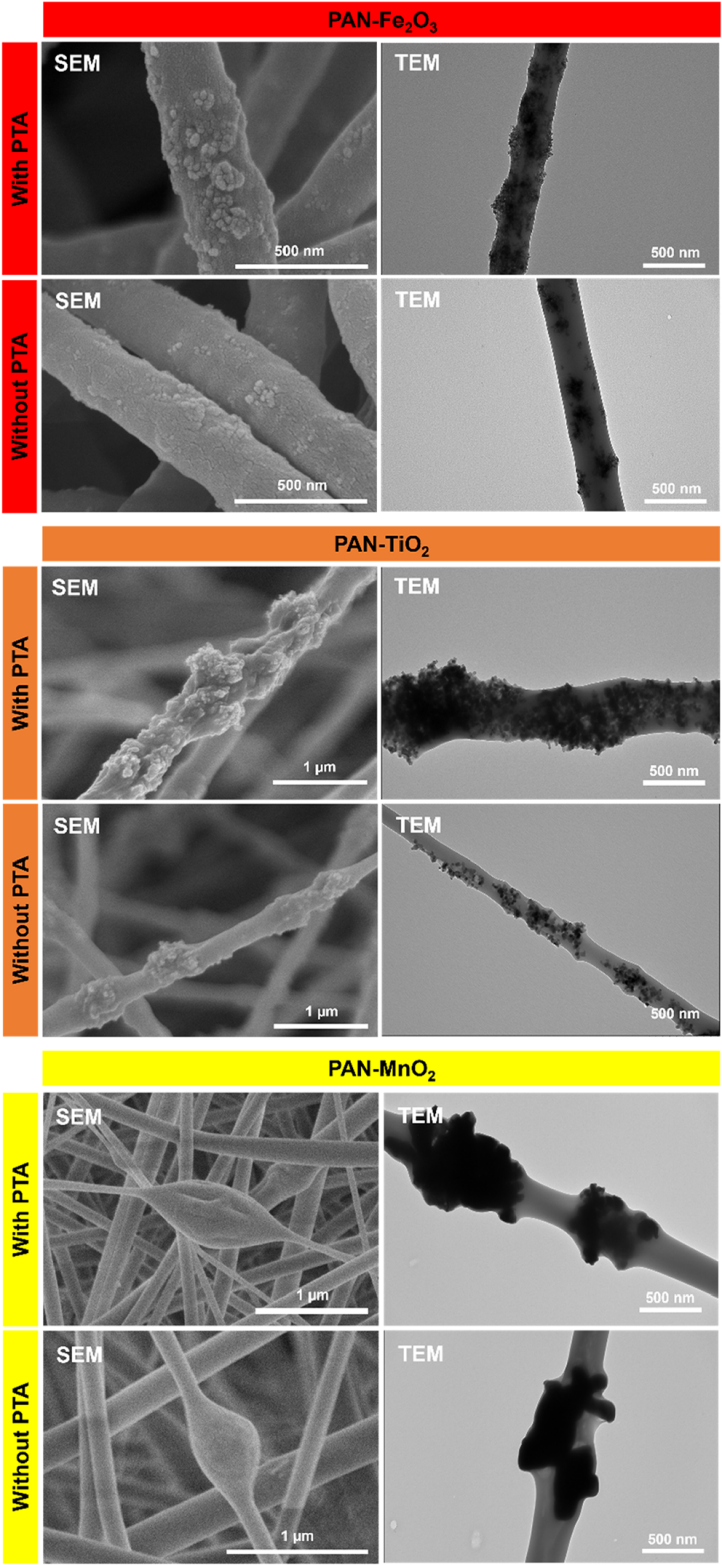
Representative SEM and TEM images of PAN-Fe_2_O_3_, PAN-TiO_2_, and PAN-MnO_2_ composite fibers fabricated either with or without the addition of PTA to the electrospinning sol- gel.

In contrast, SEM revealed bead-like domains several hundred nanometers in size on PAN–MnO_2_ fibers, corresponding to MnO_2_ aggregates visible in TEM. These aggregates likely form because the commercial MnO_2_ particles (hundreds of nm) are larger than the nanofiber wall thickness and thus tend to cluster within the polymer jet during electrospinning. The smooth, bulb-like appearance in SEM suggests that the polymer matrix covers these aggregates, giving the impression of encapsulation, rather than the MnO_2_ remaining exposed on the fiber surface. Therefore, the observed morphology is best explained as polymer-embedded MnO_2_ aggregates, rather than discrete nanoparticles fully encapsulated due to interfacial interactions.

Figure 3 compares representative survey and core scan X-ray photoelectron spectroscopy (XPS) spectra for composites prepared with and without PTA for PAN-Fe_2_O_3_, PAN-TiO_2_, and PAN-MnO_2_ nanofibers. The Fe 2p region of PAN-Fe_2_O_3_–PTA and Ti 2p of PAN-TiO_2_–PTA showed a significant and clear increase in Fe and Ti surface concentrations, respectively, relative to composites prepared without PTA. XPS analysis, therefore, supports the surface enrichment of iron and titanium oxide nanoparticles through the integration of PTA during synthesis. We hypothesize that this increase in Fe_2_O_3_ and TiO_2_ surface concentration results from the ability of PTA to improve nanoparticle dispersion in the sol–gel. We further hypothesize that dispersed nanoparticles (and smaller nanoparticle aggregates) are more likely to locate at the polymer–air interface because of their smaller size, which confers greater mobility in the polymer under the external forces applied during electrospinning. Notably, a similar increase in surface Mn concentration was not observed for PTA-containing composites prepared with MnO_2_. In fact, both composites prepared with and without PTA generated little to no signal in the Mn 3s and Mn 2p regions, once again suggesting that the MnO_2_ particles were most likely embedded within the polymer, although we cannot rule out that the embedded MnO_2_ particles were present in too low of an abundance to be detected by XPS.

**Figure 3:**
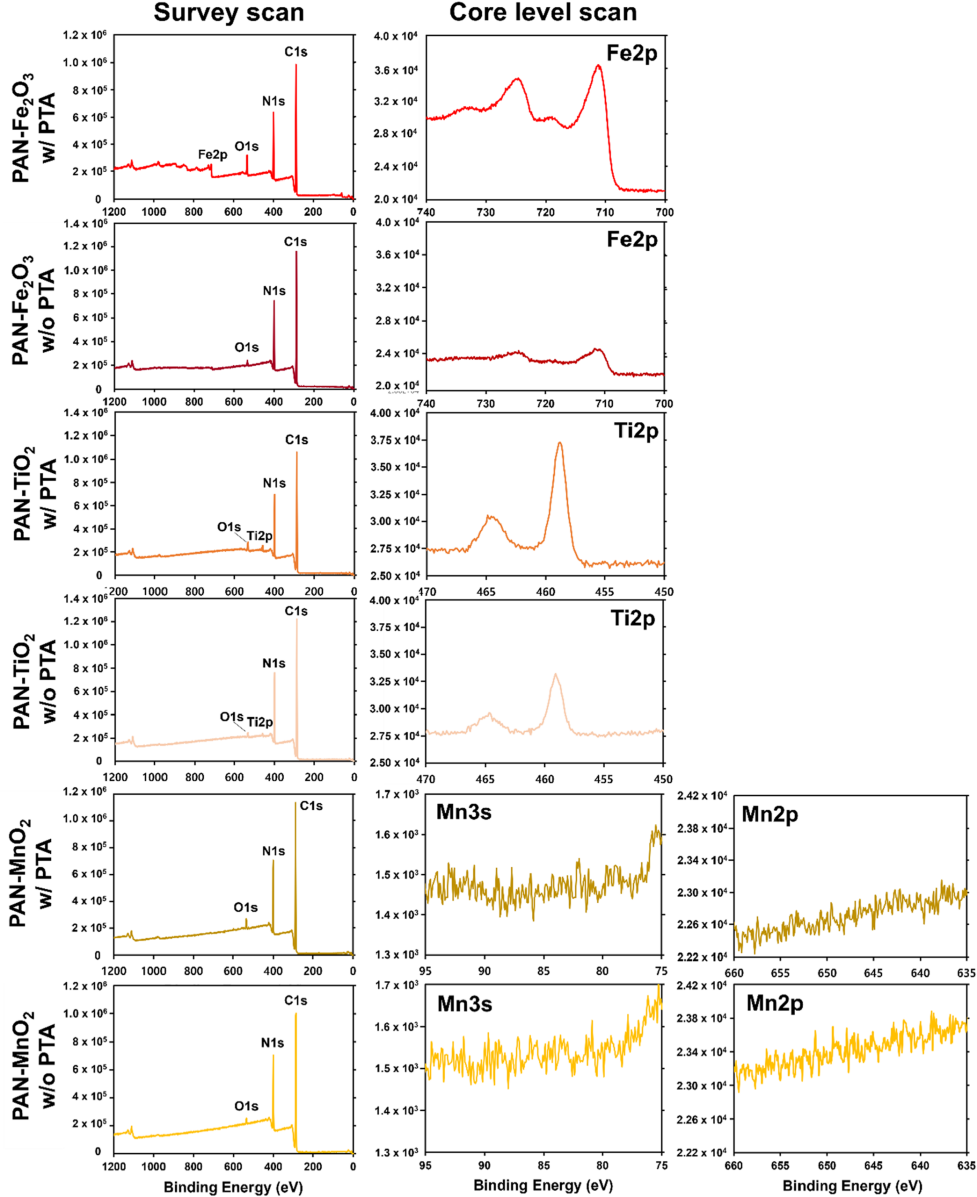
XPS survey scans and core level scans of the Fe 2p region for PAN-Fe_2_O_3_, the Ti 2p region for PAN-TiO_2_, and Mn 3s and Mn 2p region for PAN-MnO_2_. Spectra are shown for composites prepared with and without the addition of PTA to the electrospinning sol–gel.

Specific surface areas (m^2^/g) and pore volumes (cm^3^/g) from N_2_ Brunauer–Emmett–Teller (BET) analysis for metal oxide particle and composites prepared with and without PTA are provided in Figure S6. For PAN-Fe_2_O_3_, PAN-TiO_2_, and PAN-MnO_2_ synthesized without PTA, the surface area and pore volumes of composites were all comparable to those of PAN. With PTA included during synthesis, the surface area and pore volume values for PAN-Fe_2_O_3_ and PAN-TiO_2_ composites increased, often considerably. Notably, the surface area of PAN-Fe_2_O_3_–PTA more than doubled with a 4-fold increase in pore volume relative to the PAN-Fe_2_O_3_. For PAN-TiO_2_–PTA, although a more modest increase in surface area was observed relative to PAN-TiO_2_ (from about 20 to 25 m^2^/g) inclusion of PTA produced nearly a 4-fold increase in pore volume. In contrast, no increase in surface area or pore volume was observed for MnO_2_ composites prepared with PTA relative to those without PTA.

Increases in composite specific surface area are most likely from the increase in concentration of Fe_2_O_3_ and TiO_2_ nanoparticles at or near the composite surface as a result of their interactions with PTA during electrospinning. As previously shown via XPS, PTA promotes the enrichment of Fe_2_O_3_ and TiO_2_ on the composite surface, and the inherently high surface area of the nanoparticles (60 and 40 m^2^/g, respectively, for Fe_2_O_3_ and TiO_2_ nanoparticles) helps to produce more available surface area on the nanofiber surface. This is consistent with the rougher composite surfaces observed through SEM imaging.

Pore volume increases can also be attributed to PTA acting as a porogen, where the release of some of the PTA from the composite (achieved via the washing process we employ to all synthesized materials; see Methods) leaves behind open pore space. In our prior work with iron oxide nanofiber composites for lead sorption, we found that PTA was not fully retained in the fibers, producing an increase in both surface area and pore volume in washed materials.^15^ We speculate that the effectiveness of PTA as a porogen may be linked to its interactions with metal oxides, as similar increases in surface area and pore volume were not observed for PAN with PTA (see Figure S6); indeed, pore volumes for PAN with PTA were smaller than PAN without PTA, presumably from the embedded PTA blocking available pore space in the polymer. Finally, while some PTA is clearly released evidence from the metal oxide composites, we cannot rule out that a small portion of the PTA may also be retained in nanofibers and contribute to their reactivity toward U­(VI). For example, we have previously found that PAN amended with PTA exhibited greater lead uptake than PAN without PTA, suggesting that the carboxylic acid groups available in PTA can participate in lead uptake.^15^

### 3.4 Influence of PTA on U­(VI) Uptake of Metal Oxide Nanofiber Composites

For U­(VI) uptake studies, the performance of PAN nanofibers composites with different metal oxides and synthesized both with and without PTA is shown Figure 4. Results are shown from single point uptake experiments conducted with 0.25 g/L of each composite and an initial U­(VI) concentration of 1 μM at pH 7 in 5 mM CaCO_3_. Sorption of U­(VI) was determined after 24 h. Results in Figure 4a show both the mass of U­(VI) sorbed per total mass of composite at equilibrium (i.e., *q*_e_ in mg U­(VI)/g composite) and the percent of the total U­(VI) originally in the experimental system that was bound to the composites after 24 h. In Figure 4b, we present the amount of U­(VI) sorbed per unit mass of metal oxide at equilibrium (i.e., *q*_e_ in mg U­(VI)/g metal oxide) based on the known weight percent of each metal oxide in the PAN-based composite.

**Figure 4:**
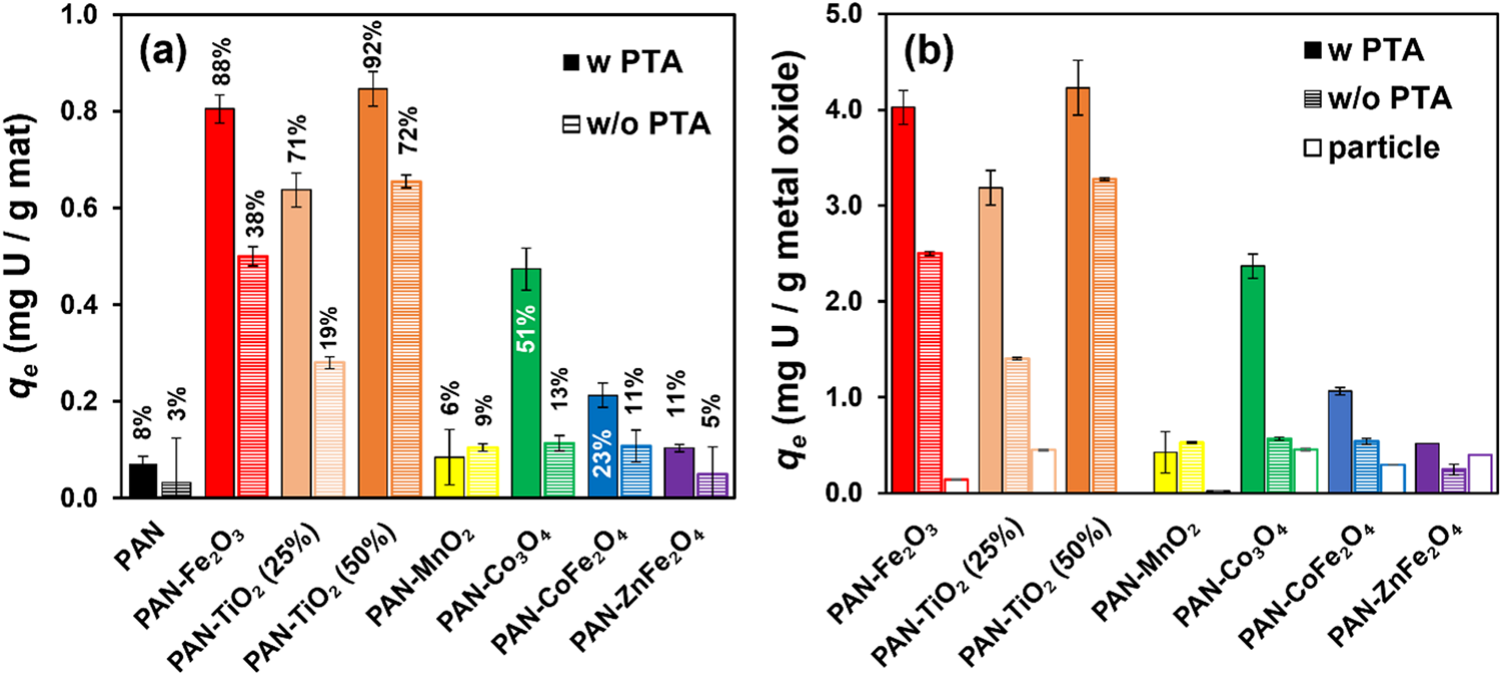
(a) Sorbed U­(VI) mass at equilibrium (*q*_e_) on PAN-metal oxide nanofiber composites with 25 wt % metal oxide (unless otherwise noted). Numbers indicate corresponding U­(VI) removal (%) based on the amount of U­(VI) remaining in solution at equilibrium relative to the total initial U­(VI) added to the system. (b) Sorbed U­(VI) mass at equilibrium (*q*_e_) normalized to the mass of metal oxide within the nanofiber composites. For comparison, the U­(VI) uptake by metal oxide particles alone (open bar) is also shown in (b). Error bars represent one standard deviation from triplicate experiments. The error bar for the particle performance in (b) is very small and may not be distinguishable. Experimental conditions: initial U­(VI) concentration of 1 μM, pH 7 (buffered with 5 mM CaCO_3_), contact time of 24 h, and nanofiber loading of 0.25 g/L.

For composites prepared without PTA, significant U­(VI) uptake was only observed for PAN-Fe_2_O_3_ and PAN-TiO_2_. For PAN-TiO_2_, we prepared two formulations at two different mass loadings of TiO_2_ in the PAN nanofibers (25% and 50% by weight relative to the initial sol–gel mass). As expected, U­(VI) sorption increased with increasing TiO_2_ mass loading, consistent with sorption occurring primarily on the surface of the embedded oxides. For all other metal oxide composites, U­(VI) sorption was comparable to unmodified PAN fibers. PAN fibers exhibited limited U­(VI) uptake (0.07 mg/g), as we previously observed in our work with amidoxime functionalized PAN, which presumably results from the interactions between U­(VI) and the –C ≡ N functionalities in the polymer backbone.^32^

Most nanofiber composites synthesized with PTA exhibited improved U­(VI) uptake relative to their corresponding composite without PTA. Consistent with observed increases in surface area and pore volume, U­(VI) sorption increased more than 2-fold for PAN-Fe_2_O_3_–PTA and more than 3-fold for PAN-TiO_2_–PTA at 25 wt % relative to the corresponding composites without PTA. In the case of PAN-Fe_2_O_3_–PTA, this increase resulted in the uptake of almost 90% of the available U­(VI) in our experimental systems, a percent removal also achieved with PAN-TiO_2_–PTA at 50 wt %. We also observed a 5-fold increase in uptake with inclusion of PTA for PAN-Co_3_O_4_, and a doubling of U­(VI) uptake for composites of CoFe_2_O_4_ and ZnFe_2_O_4_ when prepared with PTA. Uptake of U­(VI) was generally low on PAN-MnO_2_ nanofibers, and there was no significant difference in sorption of U­(VI) for MnO_2_ composites prepared with (0.18 mg/g) and without PTA (0.22 mg/g). These results are consistent with the generally poor performance of MnO_2_ particles in suspension as sorbents for U­(VI).

Based on surface-area-normalized values of U­(VI) sorption (Table S2), the greater U­(VI) uptake for Fe_2_O_3_ composites prepared with PTA (0.017 mg U­(VI)/m^2^ composite) relative to Fe_2_O_3_ composites without PTA (also 0.017 mg U­(VI)/m^2^ composite) can mostly be attributed to their higher specific surface area. In contrast, surface area normalization could not account for the increase in U­(VI) sorption observed for TiO_2_ (25 wt %) composites prepared with PTA relative to those without PTA (Table S2), but the increase in U­(VI) sorption was comparable to the increase in pore volume for TiO_2_ composites synthesized with PTA. Also, for TiO_2_ composites, although inclusion of PTA increased U­(VI) sorption in both the 25 and 50 wt % TiO_2_ composites, the greatest increase was observed for the 25 wt % composite. In fact, the 25 wt % TiO_2_ composites with PTA exhibited comparable U­(VI) uptake when compared to the 50 wt % TiO_2_ composites without PTA. These results illustrate the value of including PTA during synthesis; the improved TiO_2_ particle dispersion provided by PTA allows the embedded TiO_2_ to be more effective as a sorbent through enrichment at the composite surface. Without PTA, it requires twice as much TiO_2_ mass to achieve comparable U­(VI) sorption, presumably because a larger fraction of the TiO_2_ in the 50 wt % composite without PTA is embedded within the bulk polymer and less accessible to solution for U­(VI) binding.

Interestingly, performance trends for U­(VI) sorption on metal oxide composites did not fully match expectations from trends in U­(VI) uptake in metal oxide particle suspensions (see Figure 1). In metal oxide particle suspensions, Fe_2_O_3_ particles exhibited the second lowest U­(VI) uptake relative to all other metal oxides, only outperforming MnO_2_. In contrast, PAN-Fe_2_O_3_ composites exhibited the greatest degree of U­(VI) binding when compared to all other composites (those prepared at 25 wt %), regardless of whether PTA was included during synthesis. Similarly, suspensions of TiO_2_ nanoparticles exhibited comparable U­(VI) uptake relative to Co_3_O_4_ and the mixed metal oxides CoFe_2_O_4_ and ZnFe_2_O_4_. Although PAN-TiO_2_ composites resulted in similar U­(VI) sorption to that observed for composites with these other metal oxides when all were prepared without PTA, PAN-TiO_2_–PTA composites outperformed composites of these other metal oxides when all were prepared with PTA, especially composites of CoFe_2_O_4_ and ZnFe_2_O_4_.

We attribute differences in U­(VI) sorption on composites relative to particle suspensions to differences in the availability of reactive surface area in each sorbent system. For example, the Fe_2_O_3_ nanoparticles exhibit the smallest primary particle size and though not extensively investigated, would be expected to aggregate considerably in aqueous suspensions, thereby consuming reactive surface area. During electrospinning, we hypothesize that stabilization of Fe_2_O_3_ by PTA improves particle dispersion in the sol–gel and produces a surface-enriched composite where more reactive surface area per Fe_2_O_3_ particle is available at the solution interface for U­(VI) binding than in an aggregated Fe_2_O_3_ particle suspension.

Practically, this illustrates how the performance of polymer–metal oxide composites cannot always be generally inferred from metal oxide suspensions, and that interactions between the polymer, the metal oxides, and additives like PTA can produce composites with reactivity that is distinct or unique from its individual components. In fact, when we normalize the extent of U­(VI) sorption by the amount of metal oxide available in each composite (Figure 4b; based on their 25 wt % relative to PAN in these formulations), we find far greater removal of U­(VI) than would be predicted from the uptake we observed in metal oxide suspensions (see Figure 1). For example, in suspensions of Fe_2_O_3_ nanoparticles, we observed ∼0.13 mg U­(VI)/g Fe_2_O_3_, corresponding to uptake of just under 30% of the available U­(VI) in the system. For PAN-Fe_2_O_3_ composites, we observed ∼2.4 mg U­(VI)/g Fe_2_O_3_ for composites prepared without PTA, and ∼4 mg U­(VI)/g Fe_2_O_3_ for composites prepared with PTA. These correspond to roughly 18- and 30-fold increases in U­(VI) sorption per g of available Fe_2_O_3_. Presumably, this results from some combination of improved access of Fe_2_O_3_ to the solution when immobilized in the composite and an improved binding environment for U­(VI) near the composite surface, which we hypothesize may result from residual PTA present in the composite.

The presence of residual phthalic acid, which would be fully deprotonated as phthalate at pH 7, on the composite surface cannot outcompete the carbonate anion but could engage in cooperative binding that enhances the U­(VI) uptake when associated with certain metal oxides. The stability constants for the U­(VI) carbonate species (see Table S4) are higher than those reported for the phthalate species, indicating that ligand substitution is unlikely. However, Hwang et al.^33^ demonstrated that when the pH is between 5 and 7, phthalate binds to the Fe_2_O_3_ surface in an outersphere coordination. With this flexible coordination environment, the phthalate may be available to interact with U­(VI) through either open binding sites or through intermolecular interactions, such as hydrogen bonding, potentially enhancing U­(VI) adsorption. We cannot rule out that a similar mechanism may also be at play for some of the other metal oxides that exhibited greater uptake in the presence of PTA.

Finally, although we did not conduct extensive characterization of the other metal oxide composites, we attribute their observed increase in U­(VI) sorption when synthesized with PTA to the same phenomena responsible for increases in Fe_2_O_3_ and TiO_2_ composites. Except for MnO_2_, we presume that surface segregation of metal oxide particles was generally enhanced through the inclusion of PTA during synthesis, resulting in more metal oxide surface sites for U­(VI) uptake compared to nanofiber composites prepared without PTA. Moreover, release of some of the PTA after synthesis provides more surface area (via created pore volume) in the nanofiber composites, whereas any residual PTA may also contribute to U­(VI) uptake.

### 3.5 Effect of Initial U­(VI) Concentration on PTA-Assisted Nanofiber Composite Performance

Because of their better performance relative to other metal oxide composites, U­(VI) sorption isotherm and pH-edge experiments were only conducted with PAN-Fe_2_O_3_ and PAN-TiO_2_ composites. Sorption isotherms along with the corresponding extent of total U­(VI) removal are shown in Figure 5a,5b for PAN-Fe_2_O_3_ and PAN-TiO_2_, respectively. As a result of the wide-range of U­(VI) concentrations explored, data are shown on a log–log scale for composites made with and without PTA (see Figure S7 for raw isotherm data on nonlog scale). In all composite systems, the removal of U­(VI) was typically greater than 90%, with higher removal efficiencies approaching 100% for those composites prepared with PTA. Further, across all U­(VI) concentrations explored, composites prepared with PTA exhibited greater U­(VI) sorption. We observed only modest differences in U­(VI) uptake between PAN-Fe_2_O_3_ and PAN-TiO_2_ composites. For example, PAN-TiO_2_–PTA and PAN-Fe_2_O_3_–PTA both achieved ∼8 mg U per gram of composite at the highest initial solution phase U­(VI) concentration that we explored (2380 μg/L as U or 10 μM).

**Figure 5:**
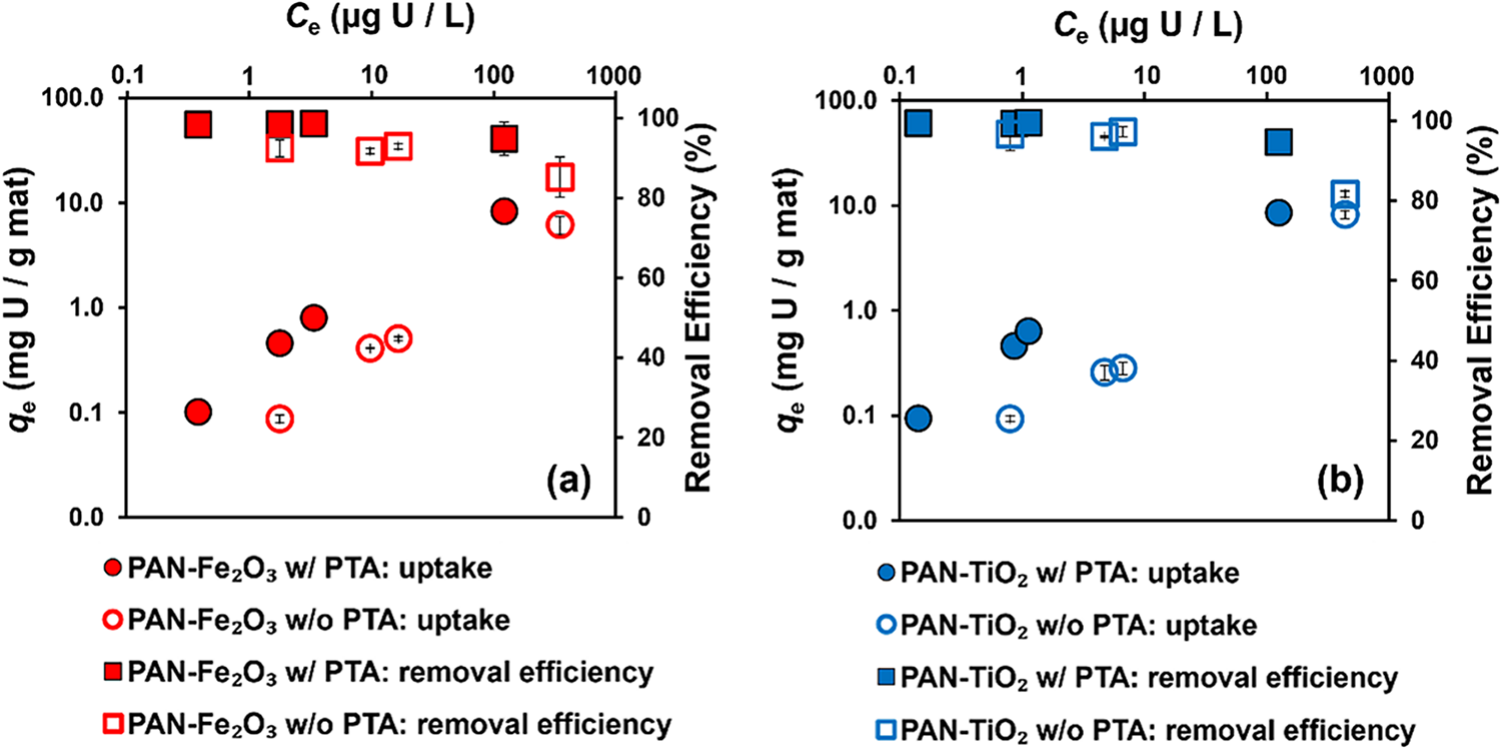
Sorption isotherms (circles, left *y*-axis) and removal efficiency (squares, right *y*-axis) of U­(VI) uptake onto (a) PAN-Fe_2_O_3_ and (b) PAN-TiO_2_ nanofiber composites (containing 25 wt % metal oxide) prepared with and without PTA. Uncertainties represent one standard deviation from triplicate experiments. Experimental conditions: initial U­(VI) concentrations of 24, 120, 240, and 2400 μg/L as uranium (0.1, 0.5, 1, and 10 μM), pH 7 (buffered by using 5 mM CaCO_3_), contact time of 24 h, and nanofiber loading of 0.25 g/L.

Generally, sorbed U­(VI) concentration increased nonlinearly as a function of equilibrium dissolved U­(VI) concentration (see Figure S7), but we observed no evidence of surface saturation across the range of U­(VI) concentrations we explored (i.e., conditions where sorbed U­(VI) concentrations were independent of solution phase U­(VI) concentration). This could simply reflect that we had not yet reached the composites’ sorption capacity at the highest initial U­(VI) concentration used in our isotherm studies [2380 μg/L as U or 10 μM U­(VI)] or that sorption capacity was reached at some point between the highest two initial solution phase U­(VI) concentrations used in isotherm experiments [between 1 and 10 μM or 238 and 2380 μg/L U­(VI)].

Because we did not observe any evidence of monolayer sorption that would be consistent with Langmuir-type adsorption, we have elected to only report results of isotherm modeling using the empirical Freundlich isotherm (eq 1)

1 $$q_{e}=K_{f}{C_{e}}^{1/n}$$

where *q*_e_ is the sorbed U­(VI) concentration at equilibrium, *C*_e_ is the dissolved, aqueous phase concentration of U­(VI) at equilibrium, *K*_f_ is the Freundlich adsorption equilibrium coefficient, and *n* is a unitless parameter that relates to the nonlinearity of the isotherm. Freundlich model best-fit parameters determined by nonlinear regression analysis are reported in Table S3. Consistent with isotherm nonlinearity, *n* values were greater than 1 for all materials, with *K*_f_ values being ∼3-fold greater for PAN-TiO_2_–PTA relative to PAN-TiO_2_, and ∼5-fold greater for PAN-Fe_2_O_3_–PTA relative to PAN-Fe_2_O_3_. We note that without clear evidence of monolayer uptake, we are unable to report a maximum concentration of sorbed U­(VI) (i.e., a sorption capacity value), which is commonly reported for newly developed sorbent materials. Instead, we suggest that our maximum observed concentration of sorbed U­(VI) (i.e., 8 mg of U per gram of composite) be used as a conservative estimate of these materials’ sorption capacity.

### 3.6 Effect of Solution pH on PTA-Assisted Nanofiber Composite Performance

The results of pH-dependent sorption experiments with PAN-Fe_2_O_3_ and PAN-TiO_2_ are shown in Figure 6, with data comparing the performance of each composite with and without PTA. As observed from sorption isotherms, composites prepared with PTA consistently outperformed those prepared without PTA across the range of pH values explored, with PAN-Fe_2_O_3_ exhibiting higher U­(VI) uptake than PAN-TiO_2_. Although uptake of U­(VI) was relatively constant across all pH values for composites without PTA, both PAN-Fe_2_O_3_ and PAN-TiO_2_ composites prepared with PTA showed significantly greater U­(VI) uptake with increasing pH.

**Figure 6:**
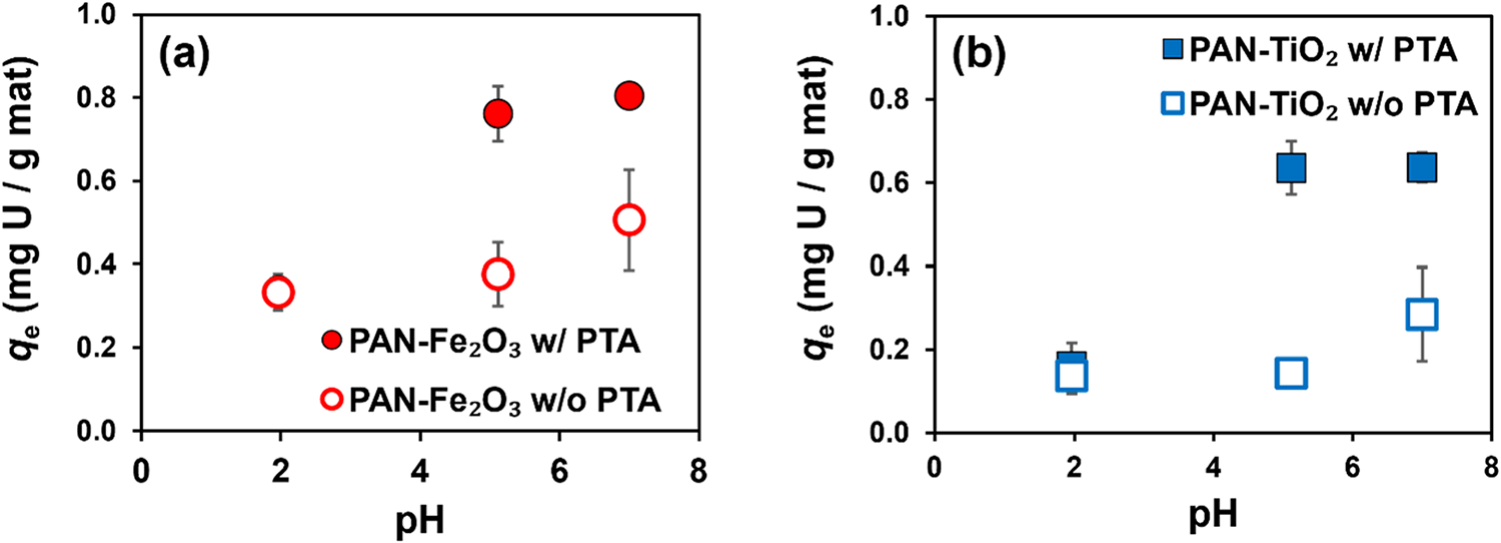
Sorbed U­(VI) mass at equilibrium (*q*_e_) as a function of solution pH for (a) PAN-Fe_2_O_3_ and (b) PAN-TiO_2_ (at 25 wt %) with or without PTA nanofiber composites. In (a), the solid and open circles are overlapping for the lowest pH value. Error bars represent one standard deviation from triplicate experiments. Experimental conditions: initial U­(VI) concentration of 1 μM, contact time of 24 h, and nanofiber loading of 0.25 g/L.

At pH 2, U­(VI) uptake was relatively low and comparable for composites both with and without PTA. From the U­(VI) speciation diagram (Figure S4), the dominant species at pH 2 is the free uranyl cation (UO_2_^2+^). Thus, a low degree of uptake would be expected at pH 2 because the surface charge of the TiO_2_ and Fe_2_O_3_ particles in the composites would also be positive. Moreover, for composites with PTA, both carboxylic acid functionalities in PTA would remain protonated and neutrally charged, as pH 2 is below the p*K*_a_ values for both groups (p*K*_a_ values of 2.9 and 5.1). Thus, they would be less likely to contribute to any U­(VI) sorption.

For PTA-containing composites, U­(VI) sorption at pH 5 increased roughly 2-fold and 3-fold relative to sorption at pH 2 for Fe_2_O_3_ and TiO_2_, respectively. At pH 5, UO_2_CO_3_(aq)^0^ becomes significant [∼25% of total U­(VI)] along with UO_2_^2+^ (∼75%) and minor contributions from [UO_2_(CO_3_)_2_]^2–^ and [Ca_2_UO_2_(CO_3_)_3_]^0^. Also at pH 5, the measured zeta potentials for both Fe_2_O_3_ and TiO_2_ were slightly negative (see Figure S3), which would help promote UO_2_^2+^ sorption. Further, one of the carboxylic acid groups in PTA would be fully deprotonated at pH 5, while the other would be partially (∼50%) deprotonated. We hypothesize that deprotonating the second carboxylate group on PTA opens up additional cooperative binding sites that are poised for U­(VI) uptake based upon the sterics of the molecule (assuming the other carboxylate group is bound to the metal oxide surface). Moreover, UO_2_CO_3_(aq)^0^ and UO_2_^2+^ have open binding sites in the equatorial plane for phthalate (i.e., full deprotonated PTA), which facilitates uptake.

Sorption of U­(VI) remained nearly constant between pH 5 and 7 for both types of composites with PTA. As previously detailed, the major aqueous U­(VI) complexes at pH 7 include [Ca_2_UO_2_(CO_3_)_3_]^0^ (∼30%) and [UO_2_(CO_3_)_2_]^2–^ (∼50%), with both [UO_2_(CO_3_)_3_]^4–^ and [UO_2_CO_3_]^0^ as additional minor species. This constitutes a shift toward more neutral to negatively charged dissolved U­(VI) species as pH increases from 5 to 7. Also with increasing pH, the surface charge of the TiO_2_ and Fe_2_O_3_ would be expected to become increasingly negative based on our zeta potential analysis, and PTA would become fully deprotonated as phthalate. Because we observed effectively the same degree of U­(VI) uptake at pH 5 and 7, we are left to speculate that cooperative binding of dissolved U­(VI) species by residual phthalate at or near the oxide surface contributes to sorption. We also cannot rule out that the chemical properties of the PTA-containing Fe_2_O_3_ and TiO_2_ composites are unique, such that U­(VI) sorption behavior cannot be easily rationalized from the expected behavior of the individual components within the composite.

### 3.7 Performance Comparison of PTA-Containing Nanofiber Composites to Other Nanofiber Sorbents Developed for U­(VI)

Several other nanofiber formulations have been developed for U­(VI) sorption. One notable study by Xie et al.^34^ used a two-nozzle electrospinning technique to fabricate mats of amidoxime-functionalized polyacrylonitrile (PAO) and poly­(vinylidene fluoride) (PVDF). This design was suggested to improve porosity and mechanical stability while retaining U­(VI) binding affinity. The resulting sorbent showed a sorption capacity of approximately 1.6 mg U/g (∼90% removal) in simulated seawater containing U, V, Fe, Co, Ni, Cu, Zn, Pb, Mg and Ca ions (experimental conditions: 0.02 g/L adsorbent dose, 24 h contact time, 25 °C, pH 5.5, and an initial U­(VI) concentration of ∼1 μM). However, the authors noted a trade-off in their approach, where lower PAO content improved surface area but reduced the number of binding sites.

In another study, Abbasizadeh et al.^35^ developed poly­(vinyl alcohol) (PVA)/TiO_2_ nanofibers modified with 3-mercaptopropyltrimethoxysilane (TMPTMS) claiming that thiol (–SH) groups would enhance U­(VI) sorption, although it should be noted that U­(VI) does not bind well to thiols (i.e., soft bases).^36,37^ The authors reported that their unmodified PVA/TiO_2_ nanofibers showed a capacity of ∼11 mg U/g while the TMPTMS-modified version reached ∼20 mg U/g (∼70% removal) (experimental conditions: 1 g/L adsorbent dose, 5 h contact time, 25 °C, pH 6, and an initial U­(VI) concentration of 30 mg/L). Although the authors attribute the improved performance to a more uniform surface and better pore structure afforded by the TMPTMS functionalization, we caution that their reported capacities may simply reflect surface precipitation rather than sorption because U­(VI) solubility at low carbonate concentrations is ∼1 mg/L at pH 6 (well below the initial concentration used in their sorption studies).^37^

As a final example, Talebi et al.^38^ synthesized electrospun nanofibers composed of PVA, sodium alginate (SA), poly­(ethylene oxide) (PEO), and HZSM5 zeolite for the removal of U­(VI) from aqueous systems. The authors reported a maximum capacity of ∼65 mg/g (∼65% removal) (1 g/L sorbent dose, 240 min contact time, 25 °C, pH 5.5, and an initial U­(VI) concentration of 100 mg/L), which they attributed to the high surface area (54.2 m^2^/g) and presence of surface functionalities such as hydroxyl (−OH) and carboxyl (−COOH) groups within the nanofiber matrix. Once again, we caution that the conditions used in these uptake experiments, most notably the lack of added carbonate to improve U­(VI) solubility, likely resulted in U­(VI) hydrolysis, nucleation, and growth of surface precipitates on their nanofibers rather than sorption processes.

In relation to these other materials, we contend that our PAN/Metal Oxide/PTA nanofibers demonstrate comparable, if not superior, performance, while offering the advantage of a simple single-step (i.e., “one pot”) synthesis that increases material viability for translation to large-scale treatment and remediation applications. Although some of the aforementioned studies with electrospun materials reported significantly higher U­(VI) uptake than we report, it is difficult, if not impossible, to compare the sorption performance of these materials to those fabricated herein given the high likelihood of U­(VI) uptake being driven by surface precipitation (and not sorption) in these earlier works. While promising as sorbents for U­(VI), we acknowledge that additional studies are needed to evaluate the performance of PTA-containing metal oxide composites in more complex water matrices containing potential competing cosolutes (e.g., organic matter, hardness causing ions, other metals), while also assessing their performance lifetime and regeneration.

## 4 Conclusion and Implications

Herein, we used electrospinning to fabricate polymer–metal oxide nanofiber composites and evaluated their U­(VI) sorption performance, both with and without incorporation of PTA. We examined a range of commercially available metal oxides including Fe_2_O_3_, TiO_2_, MnO_2_, Co_3_O_4_, CoFe_2_O_4_, and ZnFe_2_O_4_, which in aqueous suspension exhibited varying surface chemical properties and U­(VI) sorption behaviors. In nearly all instances (except for MnO_2_), composites synthesized with PTA demonstrated significantly enhanced U­(VI) uptake. Among the metal oxides considered, Fe_2_O_3_ and TiO_2_ exhibited the most promising performance upon integration into PAN nanofibers, even exhibiting U­(VI) uptake per gram of metal oxide that far exceeded metal oxide performance in particle suspensions.

The enhanced performance of composites prepared with integrated PTA is attributed to a combination of mechanisms. First, interaction of PTA with the metal oxide surface in the electrospinning sol–gel promotes particle dispersion (i.e., PTA serves as a capping ligand that limits aggregation), and this in turn results in a more uniform distribution of nanoparticles within the electrospun polymer. Second, although the mechanism is not fully understood, PTA facilitates the enrichment of metal oxides at the polymer surface (i.e., surface segregation) as supported by XPS analysis. This could simply result from the ability of smaller, better dispersed particles to move to the polymer surface from the whipping action of the polymer jet ejected from the Taylor cone during electrospinning. Alternatively, it could relate to minimizing the free energy of the PTA-capped particle within the polymer, akin to the mechanism attributed to the surface segregation of quaternary ammonium surfactants (and other charged moieties) during electrospinning.^16,39^ Third, we observed increases in both surface area and pore volume in PTA-integrated composites, consistent with PTA acting as a porogen (i.e., its release creates pores in the composite) to some degree. Fourth, some amount of PTA is likely retained in the polymer, helping to produce a better surface chemical environment through its electron rich carboxylic acid groups that can promote cooperative binding that enhances U­(VI) uptake.

Beyond fundamental insights, our results highlight the practical promise of PTA-functionalized nanofiber composites for U­(VI) remediation in aqueous environments. PTA-containing iron and titanium dioxide composites achieved >90% U­(VI) removal across a broad range of U­(VI) concentrations (up to 10 μM or 2380 μg/L) and at circumneutral pH, using relatively low sorbent loadings and embedded oxide mass. Given their electrospun structure, these composites can serve as multifunctional depth filters capable of simultaneous removal of dissolved contaminants like U­(VI) and suspended particulates, an important consideration for real-world water systems where uranium may coexist with colloidal matter.

More broadly, this work underscores two generalizable insights for advancing nanomaterial-enabled environmental technologies. First, we have found that surface chemical properties that influence particle dispersion and can be modulated through additives like PTA may matter more than nominal particle size in determining application performance of composites built with engineered nanomaterials. Second, we emphasize the value of simple, scalable fabrication strategies—like our one-pot electrospinning method—for designing multifunctional materials. PTA serves here not only as a functional additive to improve sorbent efficiency but also as a means to simplify the fabrication process by removing the need for postsynthesis modifications.

There are also broader implications of this work beyond water treatment. Given the selectivity and efficiency of these composites for uranium, they hold promise for resource recovery applications such as uranium harvesting from groundwater or waste streams associated with mining and nuclear fuel processing. Additionally, their strong sorption capabilities and environmental stability make them attractive candidates as membrane technologies for deployment in remediation of contaminated sites, especially those affected by legacy nuclear activities.

Future work should prioritize evaluation of these materials under more complex water chemistries (e.g., competing ions and natural organic matter) representative of their use in water treatment, the regeneration and reuse of these materials for applications focused on U­(VI) recovery, and the exploration of more mechanically robust polymers that could improve compsosite durability and manufacturability for such applications. For instance, transitioning this strategy to electrospun nylon could further enhance the feasibility of field deployment in rollable or modular filter configurations. Together, these findings and forward-looking directions highlight the potential of PTA-functionalized PAN/metal oxide nanofibers to contribute meaningfully to sustainable and scalable uranium remediation and recovery technologies.

## Supplementary Material



## Notes

Chemicals, characterization, analytical methods, properties of applied metal oxide particles, surface-area-normalied U upatke results, Iostherm parameters, binding constant for uranyl, PXRD patterns, TEM images and size distribution of metal oxide particles, zeta-potential of metal oxide particles, U­(VI) sepciation, digital images of nanofiber mats and electrospinning precursor, textural properties, and isotherms plot (PDF)
