# Supplementary material for: Removal of Uranium by Polymer Metal Oxide Nanofiber Composites: Enhanced Performance through Integration of Phthalic Acid
Source: ACS Appl Eng Mater. 2025 Oct 17;3(11):3920–31. doi: 10.1021/acsaenm.5c00648 (PMC12670384; doi:10.1021/acsaenm.5c00648)
Supplement: Supplementary file 1 [file em5c00648_si_001.pdf]

Supporting Information

Removal of Uranium by Polymer Metal Oxide Nanofiber Composites: Enhanced Performance through Integration of Phthalic Acid

Sewoon Kim,[a] Sarah K. Scherrer,[b] Nicole M. Shapiro,[b] Chang Min Park,[c] Tori Z. Forbes,[b] David M. Cwiertny[a,b]*

[a] Department of Civil and Environmental Engineering, University of Iowa, Iowa City, IA 52242, USA

[b] Department of Chemistry, University of Iowa, Iowa City, IA 52242, USA

[c] Department of Civil and Environmental Engineering, Kyungpook National University, 80 Daehak-ro, Buk-gu, Daegu 41566, Republic of Korea

**Corresponding author:*
E-mail: david-cwiertny@uiowa.edu *(D.M. Cwiertny)*

I. Materials and Methods

S1.1. Chemicals

Six different commercially available metal oxides were used and summarized their size and vendor in Table S1: iron oxide, titanium dioxide, manganese oxide, cobalt oxide, cobalt ferrite, and zinc ferrite. All nanofibers were prepared from the polymer polyacrylonitrile (PAN; MW 150,000) and using N,N-dimethylformamide (DMF; 99.8%) as a solvent. During electrospinning, ortho-phthalic acid (PTA; $\geq$ 99.5%) was added to select sol gels. Uranium [U(VI)] stock solutions were prepared from a 100 ppm standard solution from inorganic ventures (Lot: N2-U666947). Aqueous buffers used 5 mM calcium carbonate ($CaCO_3$), and solution pH was adjusted with 1 M NaOH or HNO_3 to the pH value desired for U(VI) uptake experiments. For U(VI) analysis, samples were acidified by nitric acid (HNO_3; 70%). All chemicals except metal oxides were obtained from Sigma-Aldrich (St. Louis, MO, USA) and used as received.

S1.2. Characterization

Powder X-ray Diffraction (XRD) with a cobalt X-ray source (MiniFlex II, Rigaku) was applied to confirm the phase of commercially available metal oxide nanoparticles. Diffraction data was collected from 10° to 80° with an interval of 0.02°.

Transmission electron microscopy (TEM; HT7800, Hitachi) and scanning electron microscopy (SEM; S4800, Hitachi) were employed to examine the morphology of metal oxide particles and electrospun nanofiber mats. For TEM and SEM sample preparation, a small amount of each particle sample was dispersed in methanol and briefly sonicated to ensure uniform

dispersion. The resulting suspension was then drop-cast onto a carbon-coated copper grid for TEM analysis, and onto carbon tape affixed to an SEM stub for SEM imaging.

Zeta potential measurements were conducted to assess the surface charge of metal oxide particle suspensions using a Nano ZS particle size analyzer (Malvern Instruments). The metal oxide particles were dispersed in deionized (DI) water at a concentration of approximately 10 mg/L and sonicated gently to ensure proper dispersion. The pH of the suspension was then adjusted gradually using 0.1 M HCl or NaOH. Zeta potential measurements were taken immediately after the pH adjustment.

X-ray Photoelectron Spectroscopy (XPS; NEXSA, ThermoFisher) with an Al Kα X-ray source was applied to confirm the elemental distributions and explore metal oxide surface chemistry. XPS was used to collect full spectrum survey scans, as well as core level scans. Additionally, N_2-BET analysis (NOVA 4200e, Quantachrome) was used to estimate surface area and pore volume for the nanofiber mats. For N_2-BET analysis, all samples were degassed at 35 °C for 12h.

S1.3. Rinsing of nanofiber mats

To evaluate the retention of PTA within the composite nanofibers, we utilized a rinsing procedure previously established in our laboratory.[S1] The as-synthesized nanofiber composites were immersed in deionized (DI) water and subjected to end-over-end mixing for 12 hours. Throughout this process, the DI water was replaced with fresh DI water every 4 hours. After rinsing, the materials were either air-dried for 12 hours prior to characterization or used directly in U(VI) uptake experiments.

S1.4. Analytical Methods

The total U(VI) in samples from batch sorption studies was determined via inductively coupled plasma–optical emission spectroscopy (ICP-MS; Agilent 7900). The ICP-MS was calibrated with a single-element U standard that was obtained from Inorganic Ventures. Samples from sorption studies with metal oxide suspensions contained dispersed metal oxide powder that was removed from the sample prior to ICP-MS analysis. In this case, the metal oxide was removed via centrifugation at 8000 g for 30 min, after which an aliquot of the supernatant was transferred to a sample vial for analysis. All collected samples were acidified with 2% HNO_3 prior to analysis.

II. Supplemental Results and Discussion

Table S1. Vendors and sizes of the applied metal oxide particles

Metal oxide	Size from vendor	Vendor
α-Fe_2O_3	3 nm	Alfa Aesar
TiO_2	21 nm	Acros Organics
MnO_2	crystals or chunks	Acros Organics
Co_3O_4	< 50 nm	Sigma-Aldrich
$CoFe_2O_4$	35-55 nm	Nanostructured & Amorphous Materials, Inc.
$ZnFe_2O_4$	40 nm	Nanostructured & Amorphous Materials, Inc.

Table S2. Surface-area-normalized U uptake based on adsorption capacities from Fig. 4.

Composites		mg U / m^2 composites
Only PAN	with PTA	0.0034
	without PTA	0.0016
PAN-Fe_2O_3	with PTA	0.0170
	without PTA	0.0172
PAN-TiO_2 (25%)	with PTA	0.0234
	without PTA	0.0088
PAN-MnO_2	with PTA	0.0041
	without PTA	0.0040

Table S3. Freundlich isotherm model parameters for PAN-metal oxide nanofiber composites used in U(VI) sorption isotherm experiments (see Fig. S7). Values were determined through least squares non-linear regression analysis using Microsoft Excel.

**Composites**		**Freundlich**		
		n	$\boldsymbol{K_F}$	$\mathbf{R^2}$
PAN-Fe_2O_3	w/ PTA	1.34 ± 0.05	0.32 ± 0.02	0.9811
	w/o PTA	1.16 ± 0.03	0.07 ± 0.01	0.9843
PAN-TiO_2	w/ PTA	1.58 ± 0.10	0.64 ± 0.03	0.9551
	w/o PTA	1.46 ± 0.02	0.20 ± 0.01	0.9626

Table S4. Reported binding constants for uranyl ion with carbonate and phthalate.

Equilibrium Reaction	Binding Constant	Reference
Carbonate binding constants		
$UO_2^{2+} + CO_3^{2-} \rightleftharpoons UO_2CO_3$	log $\beta_1 \approx 9.94$	S2
$UO_2^{2+} + 2\ CO_3^{2-} \rightleftharpoons UO_2(CO_3)_2^{2-}$	log $\beta_2 \approx 16.61$	S2
$UO_2^{2+} + 3\ CO_3^{2-} \rightleftharpoons UO_2(CO_3)_3^{4-}$	log $\beta_3 \approx 21.84$	S2
Phthalate binding constants		
$UO_2^{2+} + C_6H_5C_2O_4^{-} \rightleftharpoons UO_2$–phthalate (1:1)	log K ≈ 3.5	S3
$UO_2^{2+} + 2\ C_6H_4C_2O_4^{2-} \rightleftharpoons UO_2(\text{phthalate})_2^{2-}$ (1:2)	log $\beta \approx 12.6$	S3

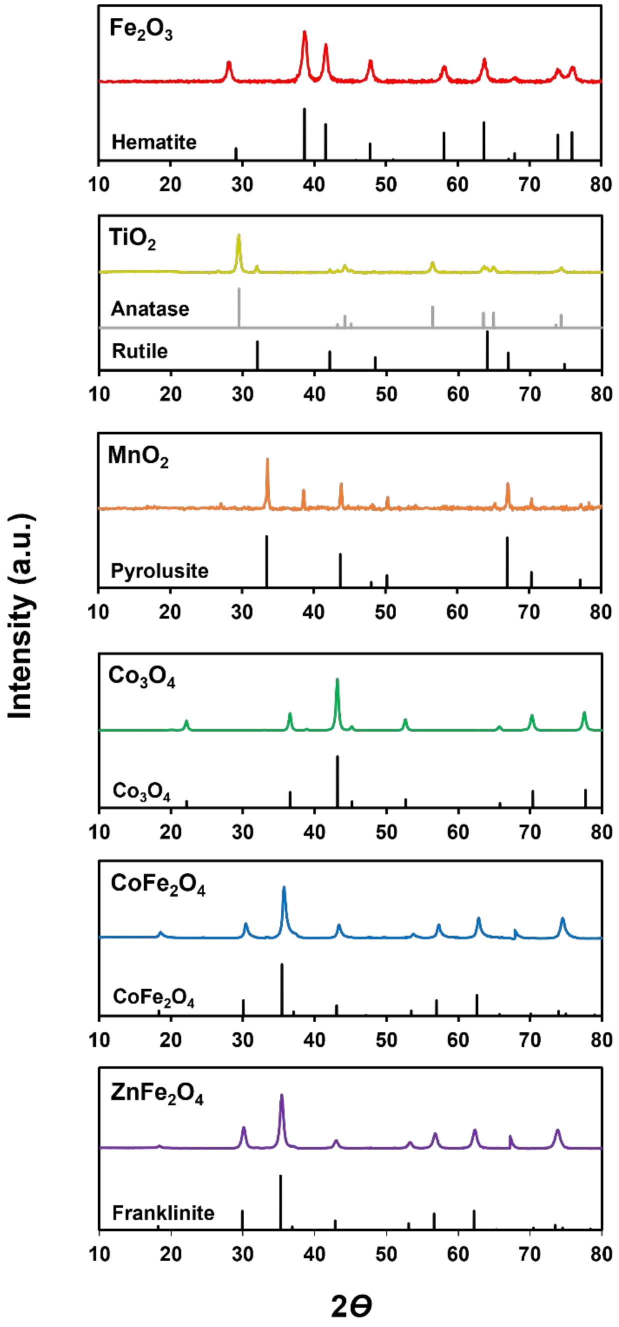

Fig. S1. Powder XRD patterns of the commercially available metal oxide particles used in this study are shown. The reference XRD patterns for Fe_2O_3, TiO_2, MnO_2, Co_3O_4, $CoFe_2O_4$, and $ZnFe_2O_4$ correspond to Hematite (98-000-0060), Anatase (TiO_2, 98-000-0008), Rutile (TiO_2, 99-000-3236), Pyrolusite (98-000-1076), Co_3O_4 (00-153-8531), $CoFe_2O_4$ (00-088-1086), and Franklinite (00-022-1012), respectively, as provided by JADE Software.

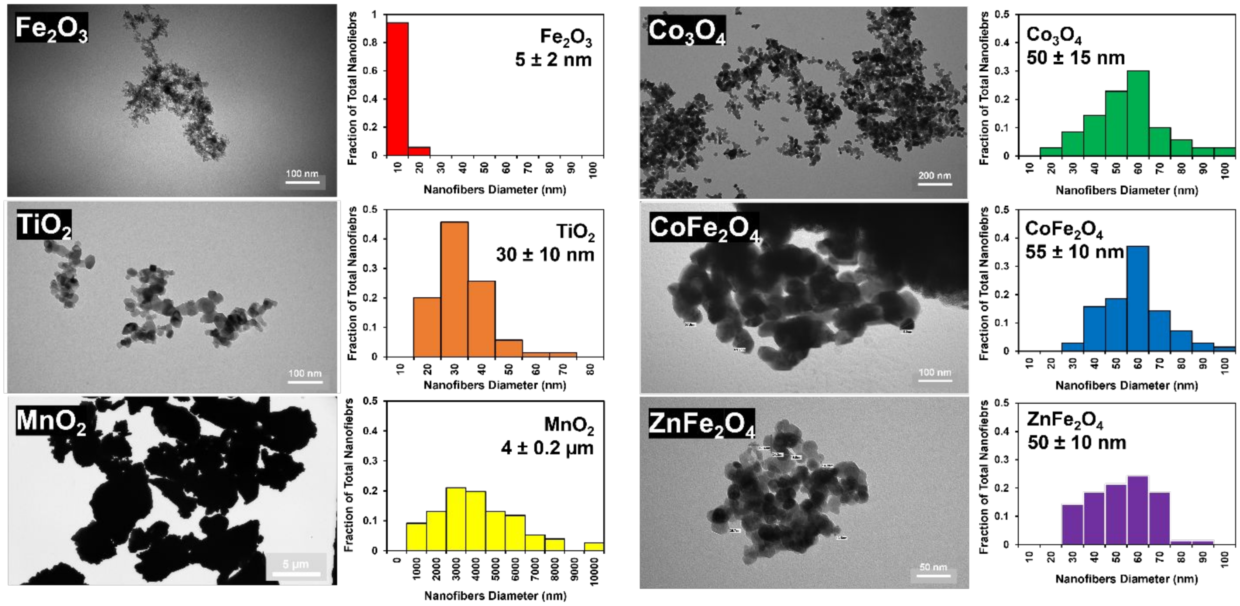

Fig. S2. TEM images and size distribution of the selected commercially available metal oxide particles were analyzed using ImageJ. The average particle size and standard deviation were determined by measuring at least $n = 100$ particles for each material, assuming a spherical shape and using the largest dimension as the primary measurement.

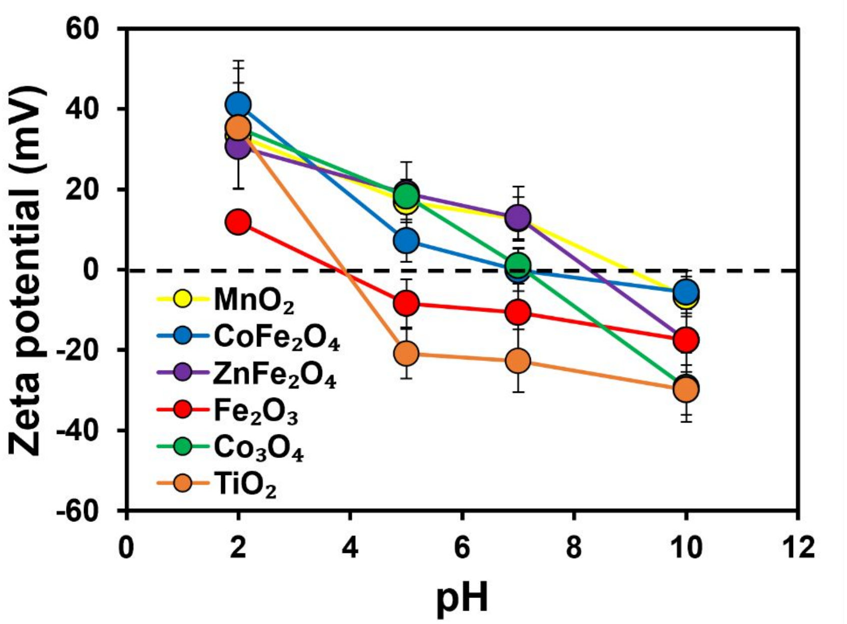

Fig. S3. Zeta Potential of selected commercially available metal oxide particles dispersed in deionized (DI) water at a concentration of 100 ppm. Measurements were conducted at 25°C, with pH adjusted using HCl and NaOH. Conductivity ranged from 0.03 mS/cm to 4.84 mS/cm. Data represent the average of three measurements, with standard deviation.

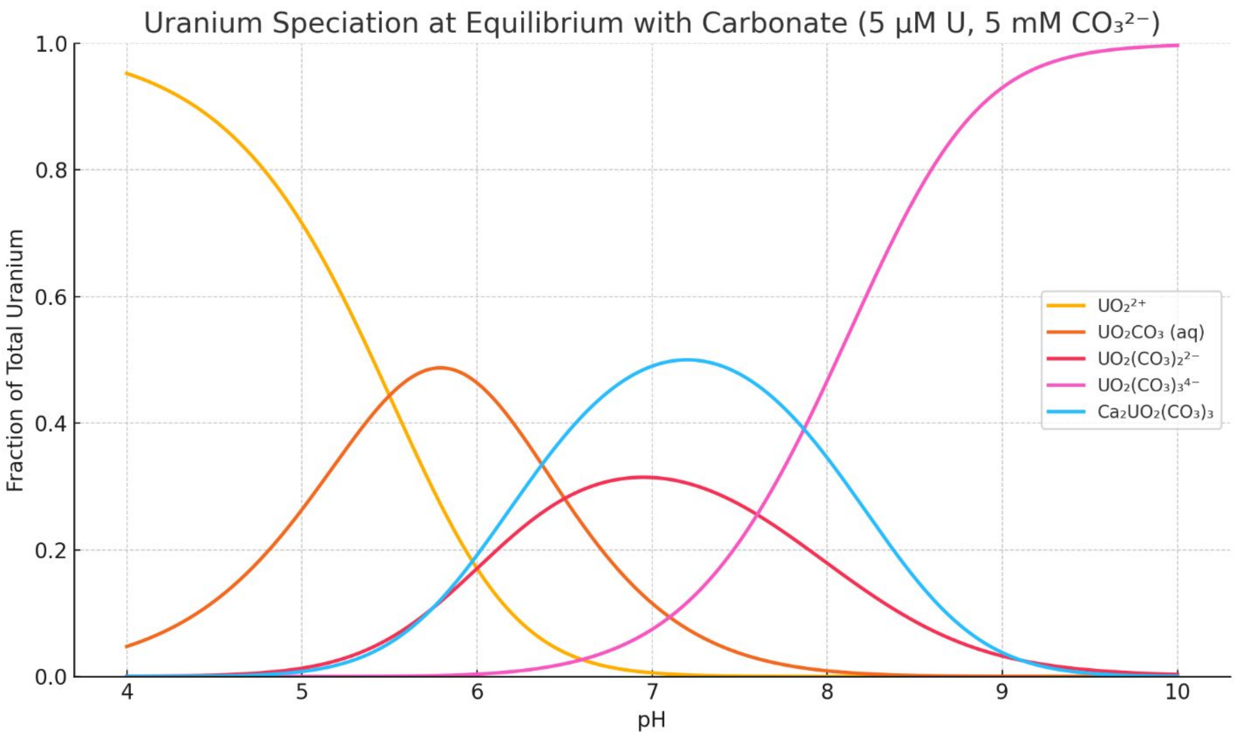

Fig. S4. U(VI) speciation diagram for 5 µM U(VI) in a 5 mM $CaCO_3$ buffer.

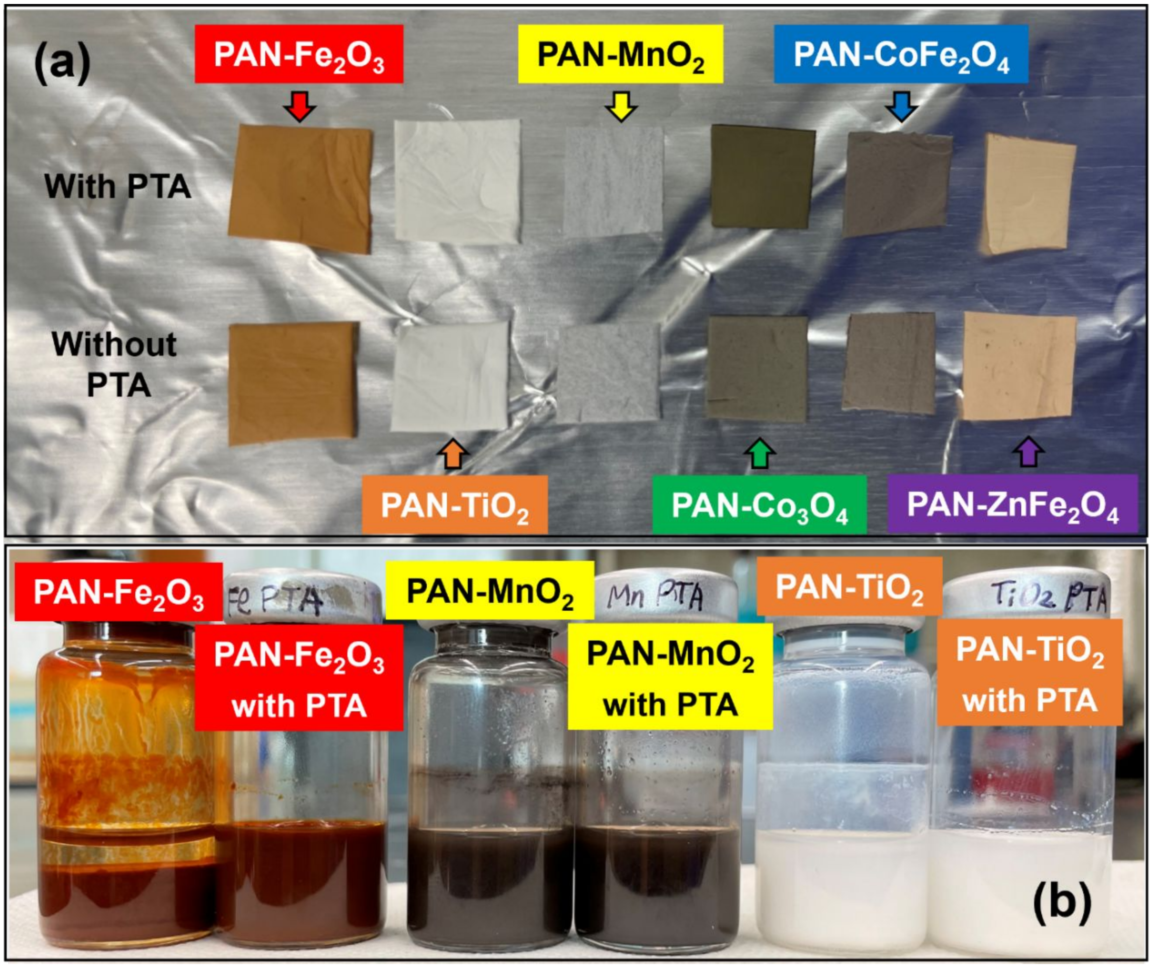

Fig. S5. (a) Digital images showing colors of different metal oxide-PAN nanofiber composites. (b) Representative digital images for precursor solution of PAN-Fe_2O_3, PAN-Fe_2O_3-PTA, PAN-MnO_2, PAN-MnO_2-PTA, PAN-TiO_2, and PAN-TiO_2-PTA after 6 h of settling.

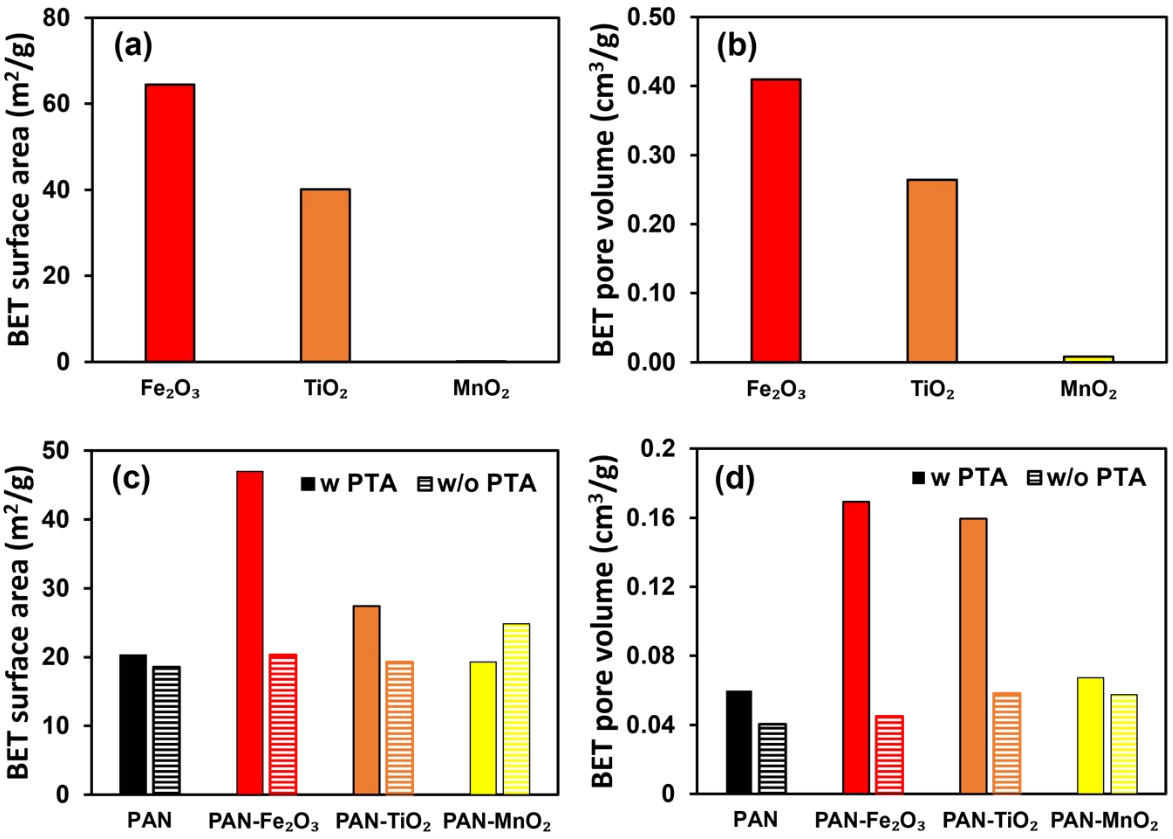

Fig. S6. (a) BET surface areas and (b) pore volumes of selected metal oxide particles. The values for MnO_2 are notably low (0.09 m^2/g and 0.008 cm^3/g, respectively), making them difficult to distinguish in the plots. (c) BET surface areas and (d) pore volumes of PAN–Fe_2O_3, PAN–MnO_2, and PAN–TiO_2 nanofiber composites, with and without PTA treatment.

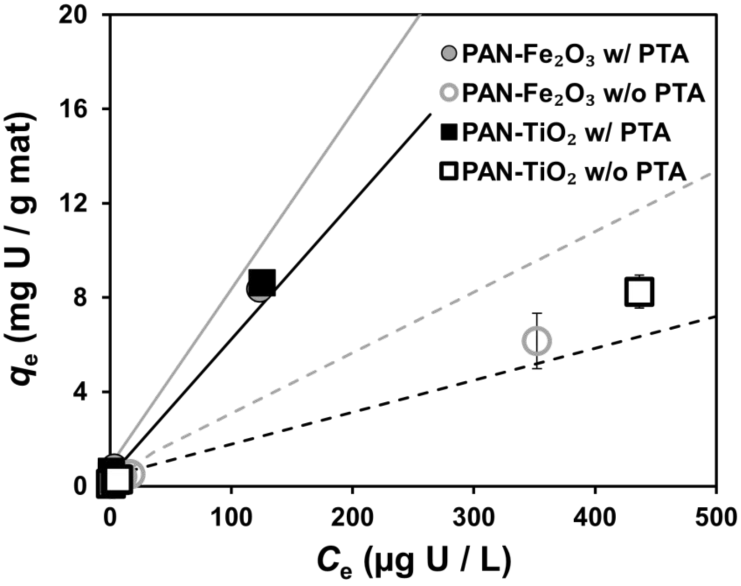

Fig. S7. Sorption isotherms for U(VI) by PAN-Fe_2O_3 and PAN-TiO_2 nanofiber composites. Lines represent Freundlich isotherm model fits determined through non-linear regression analysis. Error bars represent standard deviation from duplicate experiments. Experimental conditions: $C_{initial}$ = 24, 120, 240, and 2400 µg/L as uranium (0.1, 0.5, 1, and 10 µM); pH = 7 with 5 mM $CaCO_3$ buffer; contact time = 24 h; dosage = 0.25 g/L.

References

(S1) Kim, S.; Choi, Y.; Park, C.; Myung, N.; Cwiertny, D. Environ. Sci. Nano, 2025, **12**, 1487.

(S2) Guillaumont, R.; Fanghanel, T.; Neck, V.; Fuger, J.; Palmer, D.; Grenthe, I.; Rand, M. Amsterdam: Elsevier, 2003, **5**.

(S3) Singh, R.K.P.; Sircar, J.; Khelawan, R.; Yadava, K. Chromatographia, 1980, **13(11)**, 709-711
